# Cell-state-dependent regulation of PPARγ signaling by the transcription factor ZBTB9 in adipocytes

**DOI:** 10.1016/j.jbc.2024.107985

**Published:** 2024-11-13

**Authors:** Xuan Xu, Alyssa Charrier, Sunny Congrove, Jeremiah Ockunzzi, David A. Buchner

**Affiliations:** 1 Department of Genetics and Genome Sciences, Case Western Reserve University School of Medicine, Cleveland, Ohio, USA; 2 Department of Biochemistry, Case Western Reserve University School of Medicine, Cleveland, Ohio, USA

**Keywords:** peroxisome proliferator-activated receptor (PPAR), adipocyte, adipogenesis, E2F transcription factor, type 2 diabetes, transcriptional regulation

## Abstract

Peroxisome proliferator-activated receptor-γ (PPARγ) is a nuclear hormone receptor that is a master regulator of adipocyte differentiation and function. ZBTB9 is a widely expressed but poorly studied transcription factor that was predicted to interact with PPARγ based on large-scale protein-protein interaction experiments. In addition, genome-wide association studies (GWAS) revealed associations between *ZBTB9* and BMI, T2D risk, and HbA1c levels. Here we show that Zbtb9 deficiency in mature adipocytes decreased PPARγ activity and protein level, and thus acts as a positive regulator of PPARγ signaling. In contrast, Zbtb9 deficiency in 3T3-L1 and human preadipocytes increased PPARγ levels and enhanced adipogenesis. Transcriptomic and transcription factor binding site analyses of *Zbtb9* deficient preadipocytes revealed that the E2F pathway, controlled by the E2F family of transcription factors that are classically associated with cell cycle regulation, was among the most upregulated pathways. E2F1 positively regulates adipogenesis by promoting *Pparg* expression, independent of its cell cycle role, *via* direct binding to the *Pparg* promoter early during adipogenesis. RB phosphorylation (pRB), which regulates E2F activity, was also upregulated in *Zbtb9* deficient preadipocytes. Critically, an E2F1 inhibitor blocked the effects of *Zbtb9* deficiency on adipogenesis. Collectively, these results demonstrate that *Zbtb9* inhibits adipogenesis as a negative regulator of *Pparg* expression *via* pRB-E2F signaling. Our findings reveal cell-state dependent roles of ZBTB9 in adipocytes, identifying a new molecule that regulates adipocyte biology as both a positive and negative regulator of PPARγ signaling depending on the cellular context, and thus may be important in the pathogenesis of obesity and T2D.

Obesity is one of the greatest health challenges globally, with comorbidities including type 2 diabetes (T2D), cardiovascular disease, and certain types of cancer which collectively increase the risk of premature mortality (1). Treatments for these metabolic diseases include surgery, lifestyle modifications, and therapeutics such as the recently developed incretin mimetics (2). However, despite recent successes in the treatment of obesity and T2D, there remain many individuals for which safely maintaining a healthy body weight and long-term glucose homeostasis is a challenge, and new therapeutic approaches remain a major unmet clinical need (3, 4). Adipose tissue is the body’s primary site of fat storage and has a vital role in integrating and communicating metabolic signals. Adipose tissue expands when energy intake exceeds energy expenditure, storing the excess nutrients in lipid droplets as inert triacylglycerol (TAG), thereby maintaining metabolic homeostasis (5). Adipocytes within adipose tissues store excess energy *via* hyperplasia, an increase in the number of adipocytes, and hypertrophy, an increase in the size of adipocytes (6). Discovering the mechanisms regulating adipocyte function and adipocyte differentiation is central for understanding the pathophysiology of obesity and its related metabolic diseases and identifying new therapeutic opportunities (7, 8, 9).

Peroxisome proliferator-activated receptor γ (PPARγ) is a member of the nuclear hormone receptor family of ligand-inducible transcription factors (10). PPARγ is a master transcriptional regulator of adipogenesis and a key regulator of gene expression in mature adipocytes (11, 12). Thiazolidinedione (TZDs), synthetic ligands of PPARγ, are activators of PPARγ with robust insulin-sensitizing activities (13). Although treatment with these compounds causes weight gain and an array of additional safety issues (*e.g.*, cardiovascular risk and fluid retention), TZDs were once widely used to treat T2D (14). A more complete understanding of PPARγ activation and signaling will potentially lead to new and improved therapies for T2D. Control of PPARγ transcriptional activity depends on multi-protein complexes containing dimerization partners and other co-regulators, which each have their own physiological effects on insulin resistance and metabolic dysfunction (15, 16, 17). For example, PPAR-gamma coactivator-1α (PGC-1α) is a PPARγ coactivator that serves as a scaffolding protein to recruit a variety of other coactivators. PGC-1α regulates the activity of PPARγ on adaptive thermogenesis and fatty acid oxidation by interacting with the PPARγ/RXRα heterodimer. This interaction stimulates the expression of UCP-1, resulting in enhanced metabolic rate and insulin sensitivity and resistance to obesity (18, 19, 20, 21). NCoR and SMRT are other examples of well-characterized PPARγ co-regulators that function to recruit histone deacetylase (HDAC) complexes, which covalently modify nucleosomes to compact DNA and repress transcription (22). In the absence of ligand, PPARγ recruits the transcriptional corepressors NCoR and SMRT to downregulate PPARγ-mediated transcriptional activity. Gene silencing of *Ncor* or *Smrt* in 3T3-L1 preadipocytes increases adipocyte differentiation (22, 23, 24). These cofactors, and many others, have specific physiological functions and differential effects on regulating the transcriptional action of PPARγ, and therefore unique non-redundant roles in lipid and energy metabolism.

Zinc finger protein 407 (ZFP407) was first identified as a positive regulator of insulin-stimulated glucose uptake *via* regulation of the PPARγ signaling pathway both *in vitro* and *in vivo* (25, 26). ZFP407 was subsequently shown to directly interact with the PPARγ/RXRα protein complex (27). To better understand the mechanism by which PPARγ activity is regulated, we focused on discovering novel proteins that interact with the PPARγ and ZFP407 transcriptional regulatory protein complex. Towards this end, a previously performed high-throughput mammalian two-hybrid analysis of transcription factor protein-protein interactions (28) identified, among many other novel interactions, that ZBTB9 directly interacted with multiple proteins critical for PPARγ signaling, including PPARγ itself, but also including ZFP407 and PGC-1β (26, 29, 30, 31, 32). Furthermore, *ZBTB9* is the closest gene to a series of SNPs that are significantly associated with Body Mass Index (BMI). The lead SNP among them, *rs11757081*, was significantly associated with BMI (*p* < 10^−15^) in a GWAS meta-analysis of over 3 million individuals (33). *ZBTB9* is also the closest gene to SNP *rs210192*, which was significantly associated with T2D risk (*p* = 4.52e^−8^) in a similar meta-analysis (33). These data suggest that ZBTB9 may play a critical role in determining an individual’s risk of developing metabolic disease and T2D.

To assess the potential role of ZBTB9 in PPARγ signaling and adipocyte biology, we generated *Zbtb9*-deficienct mouse and human adipocytes and preadipocytes and demonstrated for the first time the critical role ZBTB9 has on adipogenesis and insulin sensitivity *via* modulation of PPARγ signaling. We further show that ZBTB9 is a negative regulator of early steps in adipogenesis *via* E2F-dependent regulation of *Pparg* expression. Interestingly, in mature adipocytes, ZBTB9 is required for PPARγ signaling and insulin response *via* a different mechanism that regulates PPARγ activity and protein levels but not mRNA expression. Together, our results provide the first functional characterization of ZBTB9 in adipocytes, revealing unique cell-state-dependent regulation of PPARγ signaling in both early adipogenesis and mature adipocyte function.

## Results

### ZBTB9 positively regulates PPARγ activity in mature adipocytes

**Figure 1:**
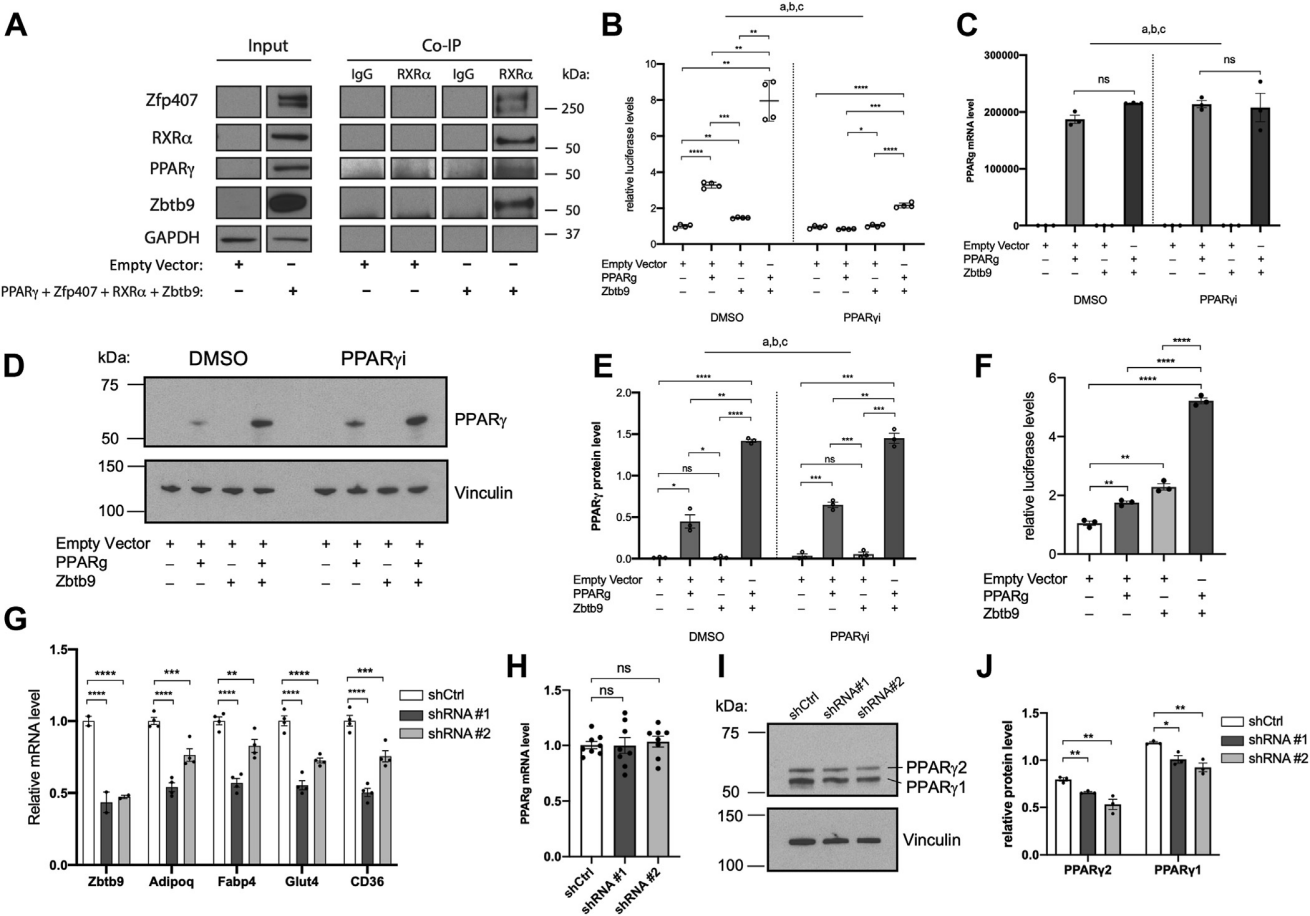
**ZBTB9 is a positive regulator of PPARγ signaling in mature adipocytes.***A*, Co-IP using anti-RXRα antibody of exogenously expressed proteins as indicated in differentiated 3T3-L1 cells. *B*, PPRE luciferase reporter activity from 293T cells transfected with an empty vector, *Zbtb9* cDNA, *Pparg* cDNA as indicated. Cells were treated with one μΜ PPARγ antagonist T0070907 (PPARγi) or DMSO as a control (Two-way ANOVA, ^a^Treatment∗overexpression *p* < 0.0001, ^b^Treatment *p* < 0.0001, ^c^Overexpression *p* < 0.0001, followed by Sídak's multiple comparisons test). *C*, *Pparg* gene expression measured by qRT-PCR and (*D*) PPARγ protein level in 293T cells transfected with plasmids as indicated (Two-way ANOVA, ^a^Treatment∗overexpression *p* not significant, ^b^Treatment p not significant, ^c^Overexpression *p* < 0.0001, followed by Sídak's multiple comparisons test). Cell lysates were subjected to SDS-PAGE and western blotting for PPARγ. Vinculin represents a loading control. *E*, PPARγ protein levels from panel D were quantified by Image J (Two-way ANOVA, ^a^Treatment∗overexpression *p* not significant, ^b^Treatment p not significant, ^c^Overexpression *p* < 0.0001, followed by Sídak's multiple comparisons test). *F*, PPRE luciferase reporter activity from differentiated 3T3-L1 adipocytes transfected with an empty vector, *Zbtb9* cDNA, *Pparg* cDNA as indicated. *G*, *Zbtb9* was knocked down in differentiated 3T3-L1 adipocytes with 2 independent shRNAs targeting *Zbtb9* (shRNA #1, shRNA #2) and compared to the control shRNA (shCtrl). Gene expression was measured by qRT-PCR. *H*, *Pparg* gene expression and (*I*) protein level (PPARγ2 and PPARγ1) in *Zbtb9*-KD 3T3-L1 mature adipocytes and control cells. Vinculin represents a loading control. *J*, PPARγ2 and PPARγ1 protein levels from panel I were quantified by Image J. ∗*p* < 0.05, ∗∗*p* < 0.01, ∗∗∗*p* < 0.001, ∗∗∗∗*p* < 0.0001, ns: not significant.

A high throughput mammalian two-hybrid analysis of transcription factor protein-protein interactions suggested that ZBTB9 interacts with PPARγ and other PPARγ-interacting proteins including ZFP407 (28). To further test whether ZBTB9 interacts with these proteins, a co-IP was performed in differentiated 3T3-L1 adipocytes. The co-IP confirmed that ZBTB9 does indeed interact with the PPARγ/RXRα/ZFP407 complex (Fig. 1*A*). To investigate the role of ZBTB9 in PPARγ signaling, ZBTB9 and PPARγ were co-expressed with a PPARγ response element (PPRE) luciferase reporter plasmid that contains 3 copies of the canonical PPARγ DNA binding sequence DR1. Together, ZBTB9 and PPARγ increased expression from the PPARγ reporter construct in both 293T cells (Fig. 1*B*) and 3T3-L1 differentiated adipocytes (Fig. 1*F*), suggesting that ZBTB9 positively regulates PPARγ activity through the canonical PPARγ response element. The increased PPARγ activity was not associated with altered PPARγ mRNA levels (Fig. 1*C*), although PPARγ protein levels were significantly upregulated by Zbtb9 overexpression (Fig. 1, *D* and *E*). When PPARγ function was blocked by the antagonist T0070907, PPARγ reporter expression was significantly reduced, however, Zbtb9 overexpression nonetheless retained the ability to increase PPARγ activity, although to a lesser extent (Fig. 1*B*). Treatment with the PPARγ inhibitor T0070907 did not affect PPARγ mRNA or protein levels, with Zbtb9 retaining the ability to increase PPARγ protein in the absence of changes to mRNA levels (Fig. 1, *B*–*E*).

To explore the role of ZBTB9 on PPARγ target gene expression under more physiological conditions, we reduced *Zbtb9* expression by lentivirus-mediated shRNA in differentiated 3T3-L1 adipocytes and observed consistently decreased expression of some well-characterized PPARγ target genes in *Zbtb9* knockdown (KD) cells compared to the control (Fig. 1*G*). Consistent with the effects on PPARγ by Zbtb9 overexpression, PPARγ mRNA level didn’t change with *Zbtb9* deficiency, but the protein level was significantly reduced (Fig. 1, *H*–*J*). To further test whether these impacts on gene expression and PPARγ protein downregulation were specifically the result of *Zbtb9* knockdown, *Zbtb9* deficient cells were transfected with a modified *Zbtb9* overexpression construct to test for phenotypic rescue. The overexpression construct was comprised of the Zbtb9 coding region with the nucleotide sequence corresponding to the regions targeted by shRNAs used in this study maximally changed at the DNA/RNA level without changing the encoded amino acid sequence. Co-transfection of this construct, but not a control empty vector, with a *Zbtb9* shRNA rescued the gene expression decreases observed during treatment of mature 3T3-L1 adipocytes with shRNA alone (Fig. S1*A*). Additionally, the decreased PPARγ protein levels caused by Zbtb9 deficiency were likewise rescued by Zbtb9 overexpression, when compared to shRNA alone and shRNA co-transfection with a control empty vector (Fig. S1, *B* and *C*). Altogether, this data suggests that ZBTB9 acts as a positive regulator of PPARγ signaling in adipocytes.

### Zbtb9 is a negative regulator of adipogenesis

**Figure 2:**
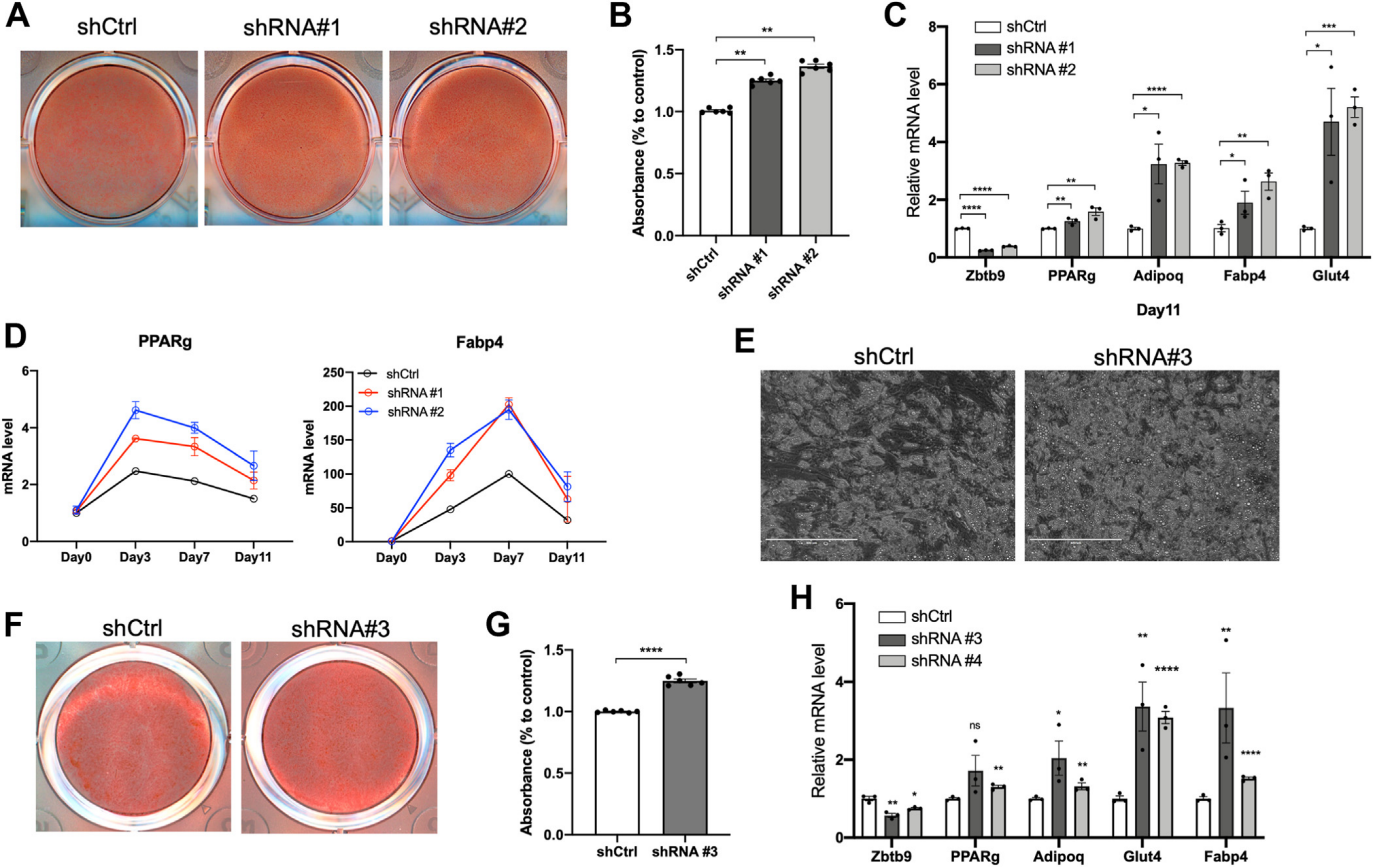
***Zbtb9* deficiency increases adipogenesis.***A*, Oil Red O staining and (*B*) quantification to assess lipid accumulation in *Zbtb9*-KD 3T3-L1 cells or control cells. *C*, *Zbtb9* and adipogenic gene expression at the end of differentiation (day 11) in *Zbtb9*-KD 3T3-L1 cells or control cells as determined by qRT-PCR. *D*, time course of *Pparg* and *Fabp4* gene expression during differentiation at D0, D3, D7 and D11 in *Zbtb9*-KD 3T3-L1 cells or control cells as determined by qRT-PCR. *E*, lipid accumulation in differentiated human adipocytes treated with *hZBTB9* shRNA (shRNA #3) or control shRNA (shCtrl) shown with brightfield images with a scale bar that indicates 400 μM and (*F*) oil red O staining and (*G*) oil red O quantification. (*H*) *ZBTB9* was knocked down with 2 independent shRNAs (shRNA #3, shRNA #4) in human preadipocytes. Gene expression was measured by qRT-PCR at the end of differentiation (day 14). ∗*p* < 0.05, ∗∗*p* < 0.01, ∗∗∗∗*p* < 0.0001, ns: not significant.

Given the interaction between PPARγ and ZBTB9 (Fig. 1*A*), the role of ZBTB9 in regulating PPARγ-dependent gene expression in mature adipocytes (Fig. 1*G*), and the central role of PPARγ in adipogenesis (31), we next examined whether ZBTB9 played a role in adipocyte differentiation. Two independent shRNAs were used to inhibit *Zbtb9* expression in 3T3-L1 preadipocytes. The cells were then induced to undergo adipocyte differentiation in the context of reduced *Zbtb9* expression. Surprisingly, lipid accumulation, as assessed by Oil Red O staining, was significantly increased in the *Zbtb9* KD cells relative to control cells (Fig. 2, *A* and *B*). Consistent with the lipid accumulation, expression of the adipogenic genes *Pparg*, *Adipoq*, *Fabp4* and *Glut4* were all significantly higher in *Zbtb9*-KD cells relative to control cells (Fig. 2*C*). The increased levels of *Pparg* and *Fabp4* expression in *Zbtb9*-KD cells were observed at different time points during differentiation, starting within 3 days of inducing adipogenesis, with bigger effects at day 3 and day 7, suggested that ZBTB9 plays an early role in regulating key genes that are critical to the molecular induction of adipogenesis (Fig. 2*D*). Analogous to the rescue experiments performed in mature 3T3-L1 adipocytes (Fig. S1, *A*–*C*), co-transfection of a *Zbtb9* shRNA with the Zbtb9 overexpression construct rescued the phenotypic consequences of shRNA-mediated Zbtb9 deficiency on adipogenesis. Zbtb9 overexpression blocked the increase in adipogenesis associated with Zbtb9 shRNA treatment, as evidenced by returning the levels of lipid accumulation (Fig. S1*D*) and gene expression (Fig. S1*E*) to control levels, further supporting the specificity of the phenotypes caused by Zbtb9 shRNA treatment.

ZBTB9 protein is highly conserved between mice and humans (77% identical at the amino acid level, Fig. S2). The level of conservation is even higher specifically within the BTB domain and the two C2H2 zinc finger domains (96% identical, Fig. S2). Thus, to extend the findings observed in mouse 3T3-L1 cells, and test whether ZBTB9 has a similar function during adipogenesis in humans, analogous studies were performed in a human-derived cell model of adipogenesis. *ZBTB9* levels were reduced by lentiviral-mediated shRNA treatment in human preadipocytes, and upon adipogenesis, there was again a significant increase in both lipid accumulation, as assessed by oil red O staining (Fig. 2, *E*–*G*) and adipogenic marker gene expression (Fig. 2*H*). Together, these data suggest that while ZBTB9 positively regulates PPARγ activity in mature adipocytes, it regulates the early stages of adipogenesis by an alternative mechanism in a manner that is evolutionarily conserved in both humans and mice. Given the impact of ZBTB9 on adipocyte differentiation, we examined its expression during adipogenesis in our experiments as well as several publicly available datasets in the Gene Expression Omnibus (GEO). *ZBTB9* was expressed at low levels throughout differentiation in both mouse and human cellular models of adipogenesis and did not demonstrate consistent regulation of its mRNA levels during this process (Fig. S3).

In complementary experiments to the KD above studies, the effects of ZBTB9 overexpression on adipogenesis and gene regulation were also tested (Fig. S4). Lentivirus that contained the *Zbtb9* coding region was used to induce overexpression in 3T3-L1 preadipocytes, resulting in ∼8-fold increase in *Zbtb9* levels (Fig. S4*A*). The cells were then induced to undergo adipocyte differentiation, however, *Zbtb9* overexpression had no significant effect on lipid accumulation or adipogenic gene expression (Fig. S4).

### Transcriptome-wide profiling reveals that Zbtb9 broadly regulates the expression of genes within the adipogenesis pathway

**Figure 3:**
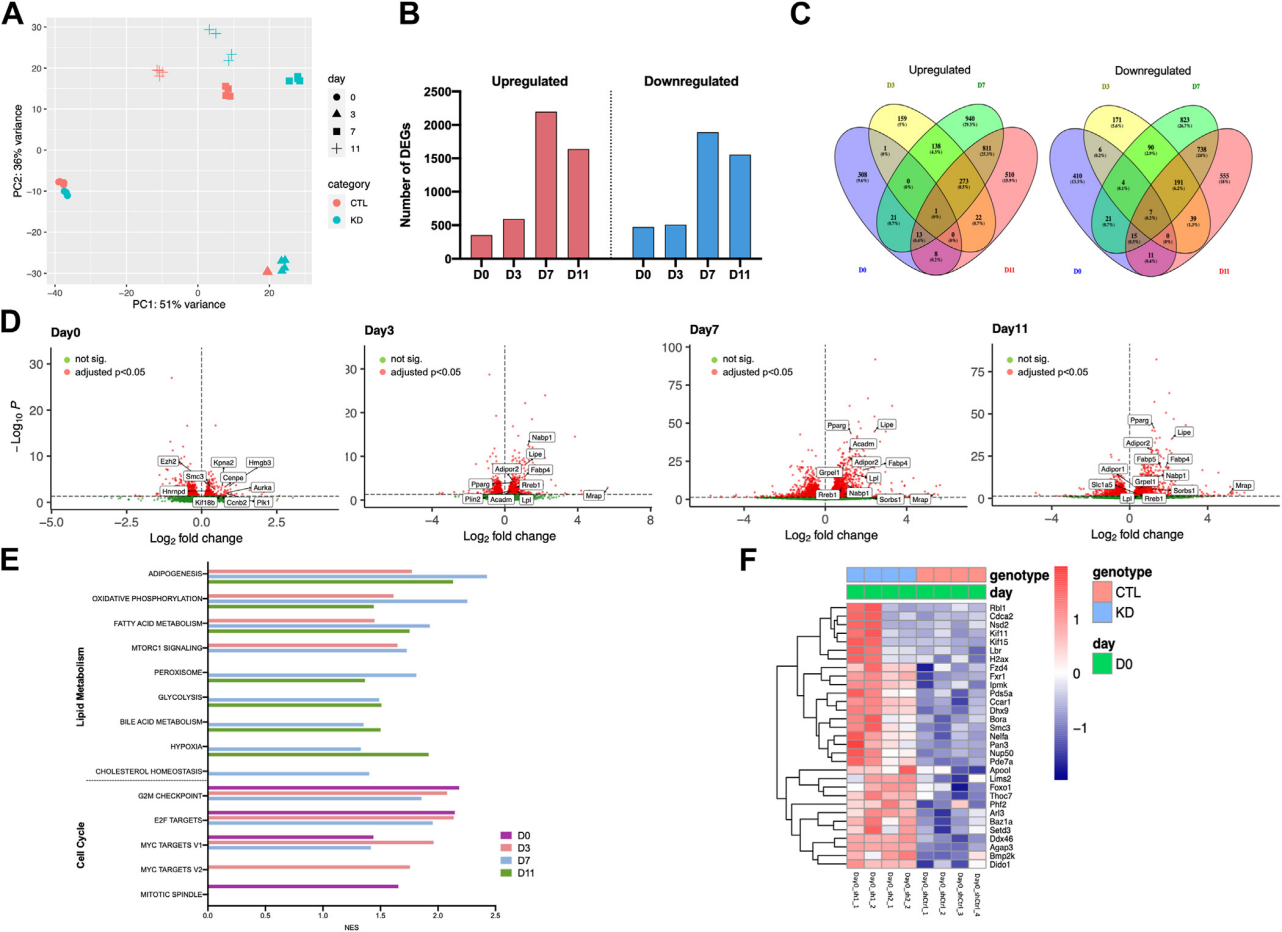
**Global transcriptional profiling reveals that *Zbtb9* regulates multiple target genes during adipogenesis.***A*, principle component analysis (PCA) of *Zbtb9* knockdown (KD) or control (CTL) transcriptomes in 3T3-L1 cells at different time points during adipogenesis. *B*, number of differentially expressed genes (DEGs) significantly (FDR < 0.05) up or downregulated upon *Zbtb9* KD in 3T3-L1 cells at D0, D3, D7, and D11. *C*, Venn diagrams showing the overlap between DEGs (*Zbtb9*-KD vs. control) upregulated or downregulated at different time points from paired comparisons. *D*, Volcano plots showing the DEGs between *Zbtb9*-KD and control 3T3-L1 cells. Highlighted are genes in E2F targets pathway (Day 0) and adipogenesis pathway (Days 3, 7, 11). *E*, gene set enrichment analysis for all DEGs using the Hallmark gene sets. All pathways shown were significantly different in *Zbtb9*-KD vs. control (FDR < 0.05). NES, normalized enrichment score. *F*, heatmap of the DEGs between *Zbtb9*-KD and control 3T3-L1 cells at day 0 that have an E2F binding motif, as predicted by TransFind. sh1_1 to 2, sh2_1 to 2, and shCtrl_1 to 4 indicate individual biological replicates of shRNA#1, shRNA#2, and shCtrl, respectively.

To identify the genes and biological processes regulated by *Zbtb9* during differentiation, *Zbtb9*-KD 3T3-L1 cells were analyzed by RNA-Seq throughout adipocyte differentiation, beginning at day 0 (before adipogenic induction), as well as days 3, day 7, and finally, day 11 when the cells are terminally differentiated into adipocytes. Principle component analysis (PCA) was performed to test whether samples clustered with each other at each time point during adipogenesis. Figure 3*A* shows the results of the PCA, demonstrating that samples at day 0 and day 3 were distinct from those at day 7 and day 11. On day 0 or day 3, there was a clear distinction between KD and control samples, whereas samples at day 7 and day 11 tended to cluster together, regardless of whether the cells were treated with a control or *Zbtb9*-targeting shRNA. This suggests that the cells have different gene profiles at later time points compared to early ones during adipocyte differentiation, consistent with the transition from preadipocytes to adipocytes. Additionally, bigger differences were observed between KD and control samples at day 7 and day 11, respectively, as compared with early time points, suggesting bigger effects of *Zbtb9* on the gene profiles later during differentiation.

**Figure 4:**
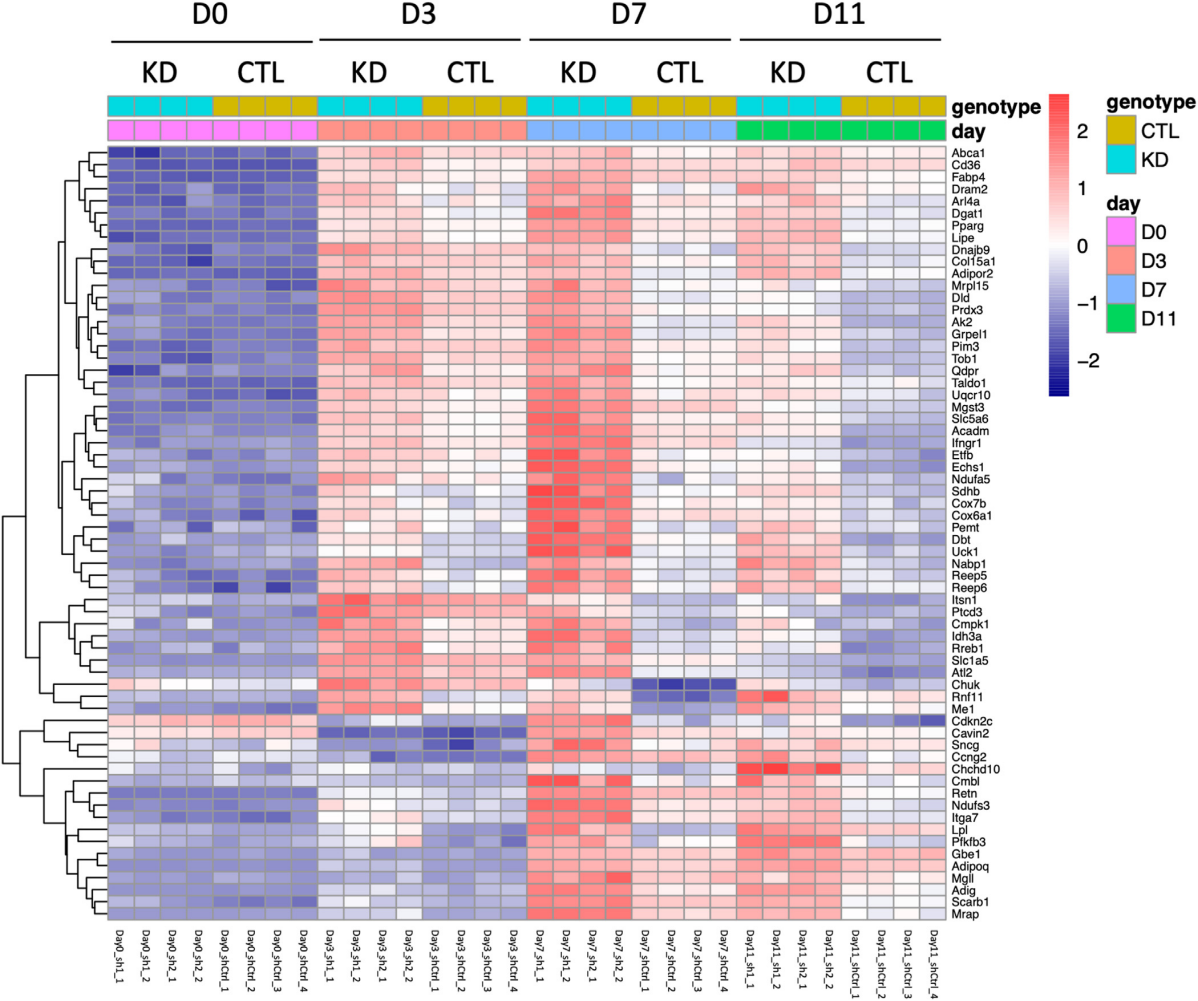
**ZBTB9 broadly regulates the expression of genes within the adipogenesis pathway.** Heatmap of the adipogenesis pathway in *Zbtb9*-KD vs. control (CTL) 3T3-L1 cells at D0, D3, D7, and D11 based on the RNA-Seq expression profiles. All genes shown in the heatmap were significantly upregulated in KD cells compared to CTL at D3, D7, and D11. Four independent replicates for each group at each time point were analyzed, including two replicates from each of the Zbtb9 shRNAs which are indicated by sh1_1 to 2 and sh2_1 to 2, as well as four replicates from the control shRNA indicated as shCtrl_1 to 4.

Differentially expressed genes (DEGs) were identified at each time point during differentiation (FDR ≤ 0.05). In general, more DEGs were observed upon adipogenic differentiation, consistent with the PCA results. A total of 826, 1103, 4088, and 3195 genes were found to be differentially regulated, with 352, 594, 2198, and 1639 gene transcripts upregulated, and 474, 509, 1890, and 1556 genes downregulated at day 0, day 3, day 7, or day 11, respectively (Fig. 3, *B*–*D*). To validate the RNA-Seq results, qRT-PCR was performed on independent samples of *Zbtb9*-KD 3T3-L1 cells and control cells. The top upregulated and downregulated DEGs at each time point, as well as *Zbtb9* itself, were tested with 90% of genes showing the same trend (up or down) in the independent samples by qRT-PCR and RNA-Seq, supporting the validity of the transcriptome studies (Fig. S5). Gene set enrichment analysis showed that upregulated genes were enriched for lipid metabolism-related pathways, and included adipogenesis, oxidative phosphorylation, fatty acid metabolism, and mTORC1 signaling (Fig. 3*E*). Increased expression of most of the pathways in *Zbtb9*-KD cells relative to control cells was observed beginning at day 3. These expression differences are likely to be largely attributable to the upregulation of *Pparg* in KD cells compared with control cells that were also seen at this time point during differentiation (Figs. 2*D* and 4). Significantly upregulated genes in KD vs. control cells of the adipogenesis pathway at days 3, 7, and 11 included many well-known adipogenic and *Pparg* target genes including *Adipoq*, *Fabp4*, *Cd36*, and *Lpl* (Fig. 4).

In addition to the downstream effects on adipogenesis likely resulting from increased *Pparg* expression, of interest were the earliest transcriptional differences that led to the upregulation of *Pparg*. For example, at day 0, before the induction of adipogenesis, there was no difference in expression levels of *Pparg* between control and *Zbtb9*-KD cells (Fig. 2*D*) and no upregulation of genes in the adipogenesis pathway (Fig. 3*E*). Thus, an important question was how *Zbtb9* was driving the increased expression of *Pparg*, one of the earliest and most critical steps in adipogenesis (31). Pathway analysis of the DEGs at day 0, which precedes the increase in adipogenesis gene expression, revealed a significant enrichment of cell cycle-related signaling pathways, including G2M checkpoint, E2F targets, MYC targets and mitotic spindle (Fig. 3*E*). To identify the transcription factor binding sites in promoter regions of these DEGs in *Zbtb9*-KD cells *versus* control cells, TransFind (34) was used to predict the transcriptional regulators of the upregulated genes in KD cells relative to control cells at day 0, and identified an enrichment of the canonical E2F binding site in KD cells (Tables 1 and S1 for detail). The identification of E2F target genes among the most upregulated pathways and enrichment of the E2F consensus binding sites upstream of DEGs was of particular interest given that E2F has previously been shown to directly regulate the expression of *Pparg* both *in vitro* and *in vivo* (35, 36). Genes with E2F binding motifs that were identified to be altered by ZBTB9 knockdown are shown in the heatmap in Figure 3*F*.

**Table 1:** Transcription factor motifs enriched in Zbtb9 deficient preadipocytes (Day 0)

Rank	Transcription factor	TF matrix	*p*-value	FDR
1	CNOT3	V$CNOT3_01	0.000001	0.000367
2	Staf	V$STAF_01	0.000016	0.001818
3	Staf	V$STAF_02	0.000016	0.001818
4	NF-Y	V$NFY_01	0.000016	0.001818
**5**	**E2F**	**V$E2F_Q3**	**0.000016**	**0.001818**
**6**	**E2F-1**	**V$E2F1_Q3**	**0.000084**	**0.008151**
7	NRF-1	V$NRF1_Q6	0.000184	0.015589
8	CREB	V$CREB_Q2	0.00039	0.017616
**9**	**E2F**	**V$E2F_Q4**	**0.00039**	**0.017616**
**10**	**E2F**	**V$E2F_Q6**	**0.00039**	**0.017616**
**11**	**E2F**	**V$E2F_03**	**0.00039**	**0.017616**
12	ETF	V$ETF_Q6	0.00039	0.017616
13	AhR	V$AHR_Q5	0.00039	0.017616
14	MECP2	V$MECP2_01	0.00039	0.017616
**15**	**E2F**	**V$E2F_02**	**0.000801**	**0.027123**
16	AP-2	V$AP2_Q6	0.000801	0.027123
17	NGFI-C	V$NGFIC_01	0.000801	0.027123
18	NF-muE1	V$NFMUE1_Q6	0.000801	0.027123
19	YY1	V$YY1_Q6_02	0.000801	0.027123
20	c-Myb	V$CMYB_01	0.001593	0.044925
**21**	**E2F-1**	**V$E2F1_Q6**	**0.001593**	**0.044925**
**22**	**Rb:E2F-1:DP-1**	**V$E2F1DP1RB_01**	**0.001593**	**0.044925**
**23**	**E2F**	**V$E2F_Q6_01**	**0.001593**	**0.044925**

∗E2F-related motifs are highlighted in bold.

### ZBTB9 modulates RB-E2F signaling to control the early induction of adipogenesis

**Figure 5:**
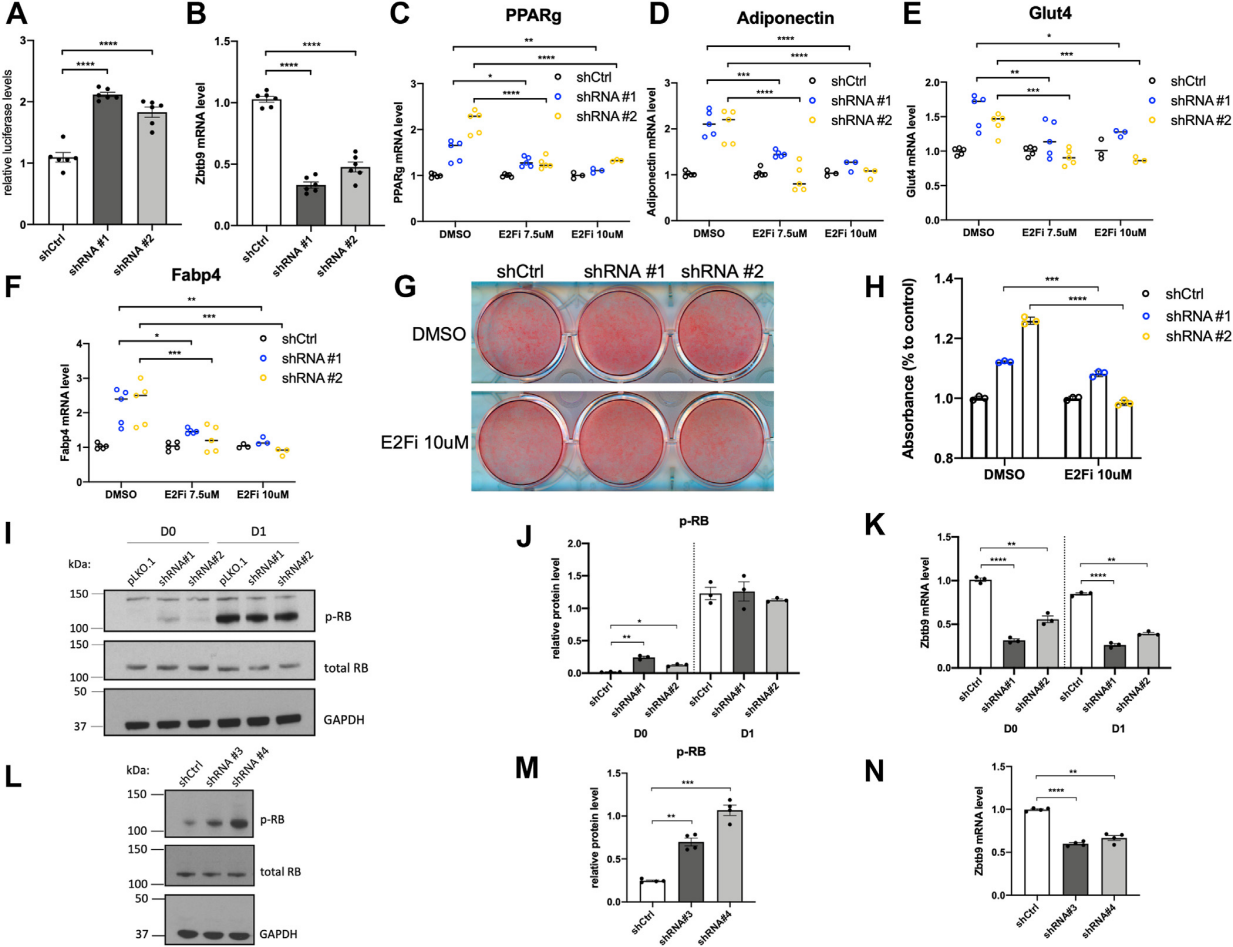
**ZBTB9 regulates early induction of adipogenesis through Rb-E2F signaling.***A*, An *E2F* consensus reporter plasmid was transfected into *Zbtb9*-KD or control 3T3-L1 preadipocytes. Luciferase activity was measured. *B*, *Zbtb9* gene expression associated with panel A. *C*–*F*, *Zbtb9*-KD or control 3T3-L1 preadipocytes were treated with the E2F inhibitor HLM006474 (E2Fi) or DMSO as a control, and differentiated. Adipogenic gene expression was analyzed by qRT-PCR at the end of differentiation (day 11). *G*, Oil Red O staining and (*H*) quantification of these cells treated with E2Fi or DMSO as a control. *I*, Western blots of *Zbtb9*-KD and control 3T3-L1 preadipocytes before the initiation of differentiation (D0) or in differentiation medium for 1 day (D1). Cell lysates were subjected to SDS-PAGE and western blotting for phosphorylated RB (p-RB) and total RB. GAPDH represents a loading control. *J*, p-RB protein levels from panel F were quantified by Image J. *K*, *Zbtb9* gene expression associated with panel F. *L*, Western blot analysis of p-RB, total RB and GAPDH (loading control) in *ZBTB9*-KD and control human preadipocytes before differentiation. *M*, p-RB protein levels from panel I were measured by image J. *N*, Human *ZBTB9* gene expression associated with panel I.∗*p* < 0.05, ∗∗*p* < 0.01, ∗∗∗*p* < 0.001, ∗∗∗∗*p* < 0.0001.

E2F is a family of transcription factors that play a critical role in early adipogenesis (37, 38). Given that *Zbtb9* deficiency causes upregulation of E2F target genes (Fig. 3*D*, Day 0), we sought to test whether the effects of ZBTB9 on adipogenesis and adipogenic gene expression were mediated by E2F. We first assessed E2F1 gene expression during adipogenesis in both 3T3-L1 and human primary preadipocytes in our data and other publicly available RNA-Seq datasets. E2F1 mRNA levels were low in human primary adipocytes and decreased during differentiation (Fig. S3, *E* and *I*). In 3T3-L1 cells, the levels of E2F1 were not consistent across experiments, occasionally decreasing as seen in human preadipocytes (Fig. S3*F*), but in most cases demonstrating no change (Fig. S3). We then examined the activity of *E2F* in 3T3-L1 preadipocytes utilizing an E2F response element luciferase reporter and demonstrated a significant increase in *E2F* activity due to *Zbtb9* deficiency (2.2- and 1.8-fold increase with shRNA#1 and #2 respectively), confirming that ZBTB9 does negatively regulate *E2F* activity (Fig. 5*A*). Next, we tested whether the increase in adipogenic gene expression and adipogenesis due to *Zbtb9* deficiency was dependent on *E2F* activity. Towards this end, *Zbtb9* was knocked down (Fig. 5*B*) in 3T3-L1 preadipocytes and the cells were induced to undergo adipocyte differentiation, either in the presence or absence of the E2F inhibitor HLM006474 (E2Fi). Consistent with previous results (Fig. 2, *A*–*C*), *Zbtb9* deficiency again increased adipogenic gene expression and adipogenesis as measured by Oil Red O lipid staining (Fig. 5, *C*–*H*). However, in the presence of E2Fi, *Zbtb9* deficiency failed to increase levels of *Pparg*, *Adiponectin*, *Glut4*, and *Fabp4*, relative to the absence of E2Fi when each of these genes was significantly increased (Fig. 5, *C*–*F*). Lipid accumulation was also significantly reduced in E2Fi-treated cells compared to DMSO control, as quantified by Oil Red O staining (Fig. 5, *G* and *H*). These results demonstrate that the effect of ZBTB9 on adipogenesis is dependent on *E2F* activity.

E2F has been shown to directly regulate the expression of lineage-specifying transcription factors, including PPARγ, both *in vitro* and *in vivo* (35, 36), which is independent of its function as a cell cycle regulator. This function of E2F is regulated by the phosphorylation of pocket protein RB (pRB), with pRB dissociating from E2F, enabling the activation and increased transcriptional activity of E2F (39). To test whether ZBTB9 regulates the phosphorylation status of RB, western blots were performed in control and *Zbtb9* KD cells in both human and mouse preadipocytes. As a control for increased pRB levels, we tested an adipogenic induction medium, which is known to induce pRB phosphorylation (38), and which indeed increased the phosphorylation of RB as expected (Fig. 5, *I*–*K*, Day 1). In addition, a clear and reproducible increase in pRB levels was detected in *Zbtb9*-KD mouse preadipocytes compared to control cells, without changing total RB levels (Fig. 5, *I*–*K*, Day 0, before induction of differentiation). In human preadipocytes, *Zbtb9*-KD also increased pRB levels at Day 0 (Fig. 5, *L*–*N*). The results demonstrated that ZBTB9 regulates adipogenesis *via* an E2F-dependent mechanism that is associated with increased pRB levels and elevated E2F activity.

## Discussion

Little is known about the cellular function of ZBTB9, although multiple GWAS suggested a role in metabolic disease susceptibility. We now report for the first time that ZBTB9 regulates adipogenesis and adipocyte function, suggesting a possible molecular mechanism underlying the altered risk of obesity and T2D associated with allelic variation near ZBTB9. We demonstrated that ZBTB9 interacts with the PPARγ/RXRα/ZFP407 protein complex in adipocytes and increased PPARγ activity *via* the consensus PPARγ/RXRα DNA binding motif. Interestingly, ZBTB9 itself modestly increased the PPARγ reporter gene expression in HEK293T cells (Fig. 1*B*), which do not express endogenous PPARγ. Thus, ZBTB9 may activate PPARγ target genes by directly binding to the response element with or without interacting with PPARγ. Unlike ZFP407, which regulates PPARγ activity in the absence of a direct effect on PPARγ levels, ZBTB9 deficiency specifically reduced PPARγ protein levels, as well as PPARγ target gene expression in 3T3-L1 adipocytes (Fig. 1).

PPARγ is important for mature adipocyte function (40, 41), and crucial for controlling gene networks involved in glucose homeostasis and insulin sensitization (31). PPARγ transactivation is induced by ligand-dependent and independent mechanisms. Ligand-dependent transactivation is induced by ligand binding to the C-terminal activation function (AF-2) domain (42). PPARs form heterodimers with RXR and bind to PPAR response elements (PPREs) in enhancers of downstream target genes (43). The binding of a ligand to PPAR results in the dissociation of a corepressor protein complex and then the recruitment of several transcriptional coactivators, some of which are responsible for the modification of histone and chromatin structure to open up DNA for transcription, while others provide linkage to core basal transcriptional machinery (44, 45). Many coactivators and corepressors of PPARγ have been reported over the past two decades, such as TIF2, PGC-1α, TRAP220/DRIP205/PBP, RIP140, NCoR, SMRT, Sirt1, and TAZ (17, 45). PPARγ and the coregulators function as multiprotein complexes to activate target gene transcription. Each of the coregulators has its own unique inherent physiological function in lipid and energy metabolism. PPARγ-mediated hormonal or non-hormonal signal transduction regulates cell growth, differentiation, development, metabolism, and other important physiological functions. An understanding of the functional significance of individual components of the complicated coregulator complexes in the PPARγ signal transduction pathway will provide multiple drug targets that may fine-tune PPARγ signaling or better integrate other signaling pathways. Our study shows that ZBTB9 functions as a positive regulator of PPARγ signaling in mature adipocytes, which adds an important piece to the PPARγ transcriptional puzzle by discovering a novel protein with its own non-redundant properties in regulating adipogenesis and adipocyte gene expression.

Adipogenesis has been studied extensively *in vivo* and *in vitro* but many questions remain about the exact molecules and mechanisms that govern this process (8, 46, 47). The growth and expansion of adipocytes and adipose tissue *in vivo* depend on the self-renewal and differentiation of adipose precursor cells (APCs), differentiation into preadipocytes, and finally the differentiation of preadipocytes into mature adipocytes (48). Adipose expansion through adipogenesis can offset the negative metabolic effects of obesity, and the mechanisms and regulators of this adaptive process are now emerging. Adipocyte differentiation involves a temporally regulated set of gene-expression events, and understanding the underlying transcriptional networks is of fundamental importance. PPARγ is the master regulator of adipogenesis (11, 12). Identifying new molecules interacting with PPARγ will shed light on the function of PPARγ in adipogenesis. One of the most consequential downstream effects of PPARγ is the activation of the transcription factor C/EBPα (49). C/EBPα and PPARγ functionally synergize to fully activate the mature adipocyte program (50, 51). Over the past two decades, many factors have been found to regulate adipogenesis. For example, transcription factor ZFP467 suppresses osteogenesis and promotes adipogenesis of the fibroblast-like progenitors by enhancing the expression of C/EBPα (52). Furthermore, KLF5 binds to and activates the *Pparg* promoter, functioning in concert with C/EBPα (53). By contrast, GATA2 and GATA3 inhibit adipogenesis through inhibition of *PPARG* transcription (54).

We further explored the role of ZBTB9 in adipocyte differentiation. Surprisingly, shRNA-mediated *Zbtb9* deficiency in preadipocytes led to an increase in adipogenesis as indicated by both lipid accumulation and adipogenic gene expression. In support of this, pathway analysis of RNA-Seq data revealed significant enrichment of genes in the adipogenesis pathway, with gene expression elevated by *Zbtb9* deficiency shortly after the induction of differentiation (Fig. 4, Day 3). These results were unexpected given the role of ZBTB9 as a positive regulator of PPARγ in mature adipocytes, for example, as illustrated in Figure 1. In contrast, PPARγ and its target genes, which are key drivers of adipogenesis, were negatively regulated by ZBTB9 at the early stages of differentiation by an alternative mechanism. Like ZFP467 and KLF5 mentioned above, the identification of ZBTB9 also helps to better define the precise mechanisms in adipogenesis, by recognizing the role of ZBTB9 and demonstrating that it works *via* the E2F pathway.

Adipogenesis involves two major events: preadipocyte proliferation and adipocyte differentiation (55). *In vitro* studies using 3T3-L1 preadipocyte model have been instrumental in studying this process. Re-entry into the cell cycle of growth-arrested preadipocytes following differentiation induction is a required initial event occurring during adipogenesis. After several rounds of clonal expansion, cells arrest proliferation again and undergo terminal adipocyte differentiation (56). E2F transcription factors can promote transcriptional activation of genes that encode cell-cycle regulators required for S-phase entry and progression of the cell cycle (57). These events are critical for mitotic clonal expansion, an obligate step in the adipocyte differentiation program (58). E2F also has important metabolic functions beyond the control of the cell cycle (59, 60, 61). For example, E2F1 was demonstrated to be a positive regulator of adipogenesis, by promoting *Pparg* expression or activity, independent of its role as a cell cycle regulator (38). Regulation of *Pparg* expression by E2F1 is through direct binding to an E2F-responsive element in the *Pparg* promoter early during adipogenesis (38). Thus, E2Fs represent a link between proliferative signaling pathways, triggering clonal expansion and terminal adipocyte differentiation through regulation of *Pparg* expression.

The pocket protein RB is a major regulator of E2F1 activity. Phosphorylation of RB results in dissociating from E2F, enabling the activation and increased transcriptional activity of E2F (39). RB has an inhibitory role at the early stage of adipocyte differentiation, through the formation of a complex including HDAC3 that inhibits PPARγ-dependent gene expression and adipocyte differentiation (62). However, the lack of RB inhibits adipogenesis in 3T3-L1 and MEF cells (63). In addition, mice with a conditional deletion of *Rb* in adipose tissue have increased mitochondrial activity resulting in an increased energy expenditure, which protects them from diet-induced obesity (64). These apparently opposite roles of RB in adipogenesis can be reconciled as during the early stage of adipocyte differentiation, cells need to exit the cell cycle. In this withdrawal stage RB plays a major role, and positively regulates adipogenesis in a PPARγ-independent manner. Later during differentiation, RB represses PPARγ activity, but the net result is still decreased fat mass in the absence of RB. Thus, the pRB-E2F1 pathway, in which we show that Zbtb9 is a key regulatory molecule, plays an important role in metabolism at different stages of adipogenesis.

The gene expression data in our study shows no difference in expression levels of *Pparg* between *Zbtb9*-KD and control cells at Day 0, which precedes the increase in adipogenic gene expression, but a significant enrichment of E2F target pathway in *Zbtb9*-KD cells compared with control cells at this time point. During this clonal expansion phase of adipocyte differentiation which represents the early stage of adipogenesis, E2F1 regulates the expression of genes implicated in the entry of the cells into the cell cycle. E2F1 also promotes *Pparg* expression at this stage, as shown by gene expression results at Day 3 (Figs. 2*D* and 4). The luciferase assay indicates increased *E2F* transcriptional activity in *Zbtb9*-KD cells, as compared to control cells, before differentiation. In line with this, RB phosphorylation was also enhanced in *Zbtb9*-KD cells at Day 0, which activated *E2F1*. Our data suggest that ZBTB9 regulates adipogenesis *via* an E2F-dependent mechanism that is associated with increased RB phosphorylation levels and elevated E2F activity.

Based on our data, ZBTB9 plays dual roles in the regulation of PPARγ signaling in a cell state-dependent manner. The mechanism by which ZBTB9 can be both a positive and negative regulator of PPARγ activity depending on the cell state remains unknown. One possibility is that there is simply little to no PPARγ present at the earliest stages of adipogenesis, and so the effects of Zbtb9 on E2F signaling are more prominent. In contrast, in mature adipocytes, when PPARγ levels are high and E2F has already played its role in triggering adipogenesis, perhaps the function of Zbtb9 to control the levels of PPARγ protein becomes more prominent. Other potential hypotheses include whether different protein interaction partners of Zbtb9 are cell-state dependent, or different post-translational modifications of Zbtb9 that are cell-state dependent, among other possibilities. Although much remains to be discovered about the underlying molecular mechanism as well as the physiological role of ZBTB9 in adiposity, our study provides new mechanistic insights into how ZBTB9 regulates early adipogenesis and adipocyte function, identifying a new molecule that may be important in the pathogenesis and treatment of obesity and T2D.

## Experimental procedures

### Cell culture

3T3-L1 cells were obtained from ATCC (#CRL-3242) and passaged and differentiated as previously described (25). Briefly, 3T3-L1 cells were induced to differentiate at day 0 (2 days post confluence) by adding the induction medium, which is the complete culture medium supplemented with the DMI cocktail (1 μM dexamethasone, 0.5 mM 3-isobutyl-1-methylxanthine, and 167 nM insulin, all from Sigma, Saint Louis, MO). At day 3, the induction medium was removed and the maintenance medium (complete culture medium supplemented with 167 nM insulin) was added. At day 7, the maintenance medium was removed, and a complete culture medium was added. The cells were harvested for staining or RNA extraction on day 11.

Human preadipocytes were obtained from Sigma (#802S-05A) and plated and cultured with Human Preadipocyte Growth Medium (Sigma #811–500). Once confluent, the cells were subjected to differentiation with Human Preadipocyte Differentiation Medium (Sigma #811D-250) for 12 days. The differentiation medium was refreshed every other day. Cells were harvested for staining or RNA extraction at the end of differentiation.

### Lentiviral production and infection

All knockdowns were performed by lentiviral-mediated shRNAs targeting either human or mouse Zbtb9 as indicated or a control shRNA. Lentiviral particles expressing either a control shRNA (shCtrl: pLKO.1, Sigma-Aldrich) or shRNAs targeting *Zbtb9* (mouse *Zbtb9* shRNA #1: TRCN0000125706; mouse *Zbtb9* shRNA #2: TRCN0000125707; human *ZBTB9* shRNA #3: TRCN0000017185; human *ZBTB9* shRNA #4: TRCN0000017186) were prepared and propagated in HEK293T cells as described previously using the second generation of psPAX2 and pMD2.G packing vectors. After two rounds of lentiviral infection, cells were selected using puromycin (2 μg/ml) and/or hygromycin B (150 μg/ml) then cultured and differentiated as previously described (32). Lentiviral vectors expressing either an empty vector vehicle (EV), the *Zbtb9* coding region, or an adjusted *Zbtb9* coding region compatible with shRNA co-transfection were synthesized by VectorBuilder Inc. (Chicago, IL) and contained an EF1A promoter as well as puromycin or hygromycin B resistance cassettes. The adjusted *Zbtb9* coding sequence contained the following modifications to nucleotides 1152 to 1176 and 1226 to 1247 of the *Zbtb9* cDNA to prevent degradation when co-transfected with Zbtb9 shRNAs #1 and #2 without changing the encoded amino acid sequence. The following sequence modifications were made with lowercase letters indicating a nucleotide altered from the original cDNA sequence (ENSMUST00000120016.3): nucleotides 1152 to 1176: 5’ - AAa CAc CAc tTg ACg GAa CAt ATG - 3’; nucleotides 1226 to 1247: 5’ - taG gCA tAT tAT GcT cAC cTT - 3’. Lentiviral particles were prepared the same way as for shRNAs. Transfection efficiency was assessed by counting viable cells after puromycin selection. Cells were stained with trypan blue (0.04%) and blindly counted as positive (dead) or negative (viable). The percentage of viable cells ranged from 93% to 98% in preadipocyte transduction, and 68% to 77% in mature adipocytes (Fig. S6).

### Oil red O staining

To make the Oil Red O (ORO) working solution, 20 ml of 0.5% of the ORO stock solution (Sigma #O1391) (in isopropanol) was added to 30 ml of deionized water. Cells were washed with PBS and fixed with 10% formalin at room temperature for 30 min, then stained with the ORO working solution for 15 min. The cells were then rinsed with water three times and then scanned with an image scanner (EPSON Perfection V600). For quantification, the stain was extracted in isopropanol and measured at 492 nm using a BioTek Epoch Reader.

### RNA analysis

RNA was collected using QIAshredder and RNeasy mini kit (Qiagen). RNA for qRT-PCR was reversed transcribed using the high-capacity cDNA reverse transcription kit without the RNase inhibitor (Applied Biosystems, Carlsbad, CA). Primer sequences are given in Table S2. qRT-PCR was performed in triplicate on a QuantStudio3 Real-time PCR system using the power SYBR Green PCR master mix (Applied Biosystems). The expression level for all genes was calculated using the ΔΔCt method relative to the *Gapdh* or *Rplp0* control gene as indicated. For RNA-Seq analysis, total RNA was isolated as described above with an additional on-column DNase treatment step. RNA quality was determined on the Agilent BioAnalyzer 2100 and all samples had an RNA integrity number score greater than 9.5. Illumina TruSeq sequencing libraries were prepared by Novogene Co., LTD using standard procedures. Samples were run on an Illumina NovaSeq generating an average of 68,511,857 reads per sample. Reads were aligned to the mouse genome (Ensembl m38.102) using TopHat version 2.1.0, SamTools version 1.3.1, and Bowtie2 version 2.2.6 (65, 66, 67). Gene expression count tables were generated using HTSeq version 0.6.1 (68) and analyzed for differential expression by DESeq2 version 1.30 (69). A false discovery rate-adjusted *p* value of < 0.05 was considered statistically significant for RNA-Seq analysis. Principle component analysis (PCA) proceeded by transforming a large set of variables (the counts for each transcript of all the samples including four KD samples and four control samples at four time points (total 32 samples)) to a smaller set of orthogonal principal components (PCs) using the plotPCA function in DESeq2. The PCA plot in Fig. 3*A* showed the sample clusters in 2D spanned by the first two principal components. The RNA-Seq expression profiling data we generated are available in the Gene Expression Omnibus series GSE253544. Publicly available gene expression data was downloaded from Gene Expression Omnibus and analyzed as described earlier from the following datasets: GSE253544, GSE95029, GSE20752, and GSE249195.

### Pathway analyses

GSEA (v.4.3.2) was performed using the Broad Institute software (https://www.broadinstitute.org/gsea/index.jsp) and the enrichment scores (default parameters) were calculated by comparing the indicated groups. The list of upregulated genes in KD cells *versus* control cells at day 0 was submitted to TransFind (34) to perform a DNA sequence motif analysis and predict the transcriptional regulators of this gene set.

### Luciferase reporter assay

To assess *Pparg* activity, HEK293T cells or 3T3-L1 mature adipocytes were transfected with DNA plasmid constructs encoding PPARγ (Addgene no. 8862), ZBTB9 (MR207329; Origene Technologies, Rockville, MD), or an empty vector control plasmid (pRK5-Myc), together with the *Pparg* target gene luciferase reporter plasmid (PPRE-X3-Tk-luc, Addgene no. 1015), and control plasmid pRL-SV40 encoding Renilla for normalization. PPARγ antagonist T0070907 was added to the cell culture 4h after transfection (1 μΜ, #10026, Cayman Chemical, Ann Arbor, MI). To assess *E2F* activity, *Zbtb9* or control shRNA treated 3T3-L1 preadipocytes were transfected with the *E2F* target gene luciferase reporter plasmid (pGreenFire1_E2F1RE, Addgene no. 112248) and control plasmid pRL-SV40 encoding Renilla for normalization. Luciferase and Renilla were measured 48 h post-transfection with the Dual-Glo Luciferase Assay System (Promega, Madison, WI). Lipofectamine 3000 (L3000015, ThermoFisher Scientific) was used for plasmid transfection in HEK293T cells and 3T3-L1 preadipocytes. Gene Pulser electroporation buffer (1,652,677, Bio-Rad) was used for plasmid transfection in mature 3T3-L1 adipocytes following the manufacturer’s instructions.

### Western blotting

Western blotting was performed and quantitated as described (25). A custom anti-ZFP407 antibody was generated in rabbits against the COOH-terminal 149 amino acids of the mouse ZFP407 protein (Proteintech Group) and has been described previously (25). The anti-ZBTB9 antibody was from Aviva Systems Biology (#ARP31669_P050). The anti-PPARγ (#2443), anti-RXRα (#3085), anti-phopho-RB (#8180), anti-RB (#9313), anti-Vinculin (#13901) antibodies were from Cell Signaling Technologies. Anti-GAPDH was from Proteintech Group (#60004–1, Rosemont, IL). The goat anti-rabbit (31,460) and goat anti-mouse (31,430) secondary antibodies were from ThermoFisher Scientific (Waltham, MA). Antibody validation for the commercial antibodies is described in detail on the respective company websites.

### Statistical analyses

All data are expressed as the mean ± SEM. Statistical significance was assessed with a two-tailed Student’s *t* test or two-way ANOVA followed by Sídak's multiple comparisons test using GraphPad Prism eight software. *p* values < 0.05 were considered statistically significant for all statistical tests.

## Data availability

All RNA-Seq data is available in the Gene Expression Omnibus at accession number GSE253544.

## Supporting information

This article contains supporting information.

## Conflict of interest

The authors declare that they have no conflicts of interest with the contents of this article.
