# Supplementary material for: Cell-state-dependent regulation of PPARγ signaling by the transcription factor ZBTB9 in adipocytes
Source: J Biol Chem. 2024 Nov 13;300(12):107985. doi: 10.1016/j.jbc.2024.107985 (PMC11681874; doi:10.1016/j.jbc.2024.107985)
Supplement: Supporting information [file mmc1.docx]

**Supporting information for**

**Cell-state dependent regulation of PPARγ signaling by the transcription factor ZBTB9 in adipocytes**

**Xuan Xu^1^, Alyssa Charrier^1^, Sunny Congrove^2^, Jeremiah Ockunzzi^2^ and David A. Buchner^1,2,*^**

^1^ Department of Genetics and Genome Sciences, Case Western Reserve University School of Medicine, Cleveland, OH, 44106, USA

^2^ Department of Biochemistry, Case Western Reserve University School of Medicine, Cleveland, OH, 44106, USA

*Corresponding author. [dab22@case.edu](mailto:dab22@case.edu).

**
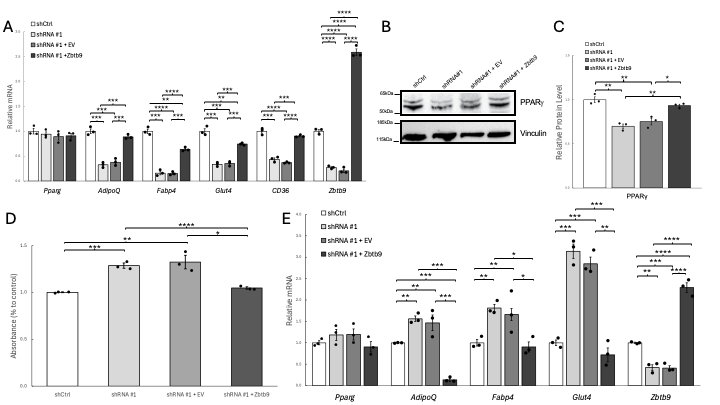
**

**Figure S1. ZBTB9 overexpression rescues phenotypic effects of shRNA knockdown in 3T3-L1 cells.** (A-C) Differentiated 3T3-L1 adipocytes were treated with a control shRNA or shRNA targeting *Zbtb9* (shRNA #1) together with overexpression of *Zbtb9* or a control empty vector (EV). (A) Gene expression changes were measured by qRT-PCR and fold change was calculated relative to the control gene *Rplp0*. (B) Protein level of PPARγ in *Zbtb9*-KD cells with *Zbtb9* overexpression or EV co-transfection in 3T3-L1 mature adipocytes was determined by western blotting. Vinculin represents a loading control. (C) PPARγ protein levels from panel B were quantified by Image J. (D,E) Adipocyte progenitors were treated with a control shRNA or shRNA targeting *Zbtb9* (shRNA #1) together with overexpression of *Zbtb9* or a control empty vector (EV) and differentiated into mature adipocytes. (D) Lipid accumulation was quantified by Oil Red O staining. (E) Gene expression changes were measured by qRT-PCR and fold change was calculated relative to the control gene *Rplp0*. For all panels * p < 0.05, ** p < 0.01, *** p < 0.001, **** p < 0.0001, only significant differences are indicated.

**Figure S2. Evolutionary conservation of the mouse and human ZBTB9 protein sequence.** Highlighted are the mouse and human ZBTB9 protein BTB domain and 2 zinc finger C2H2 domains.


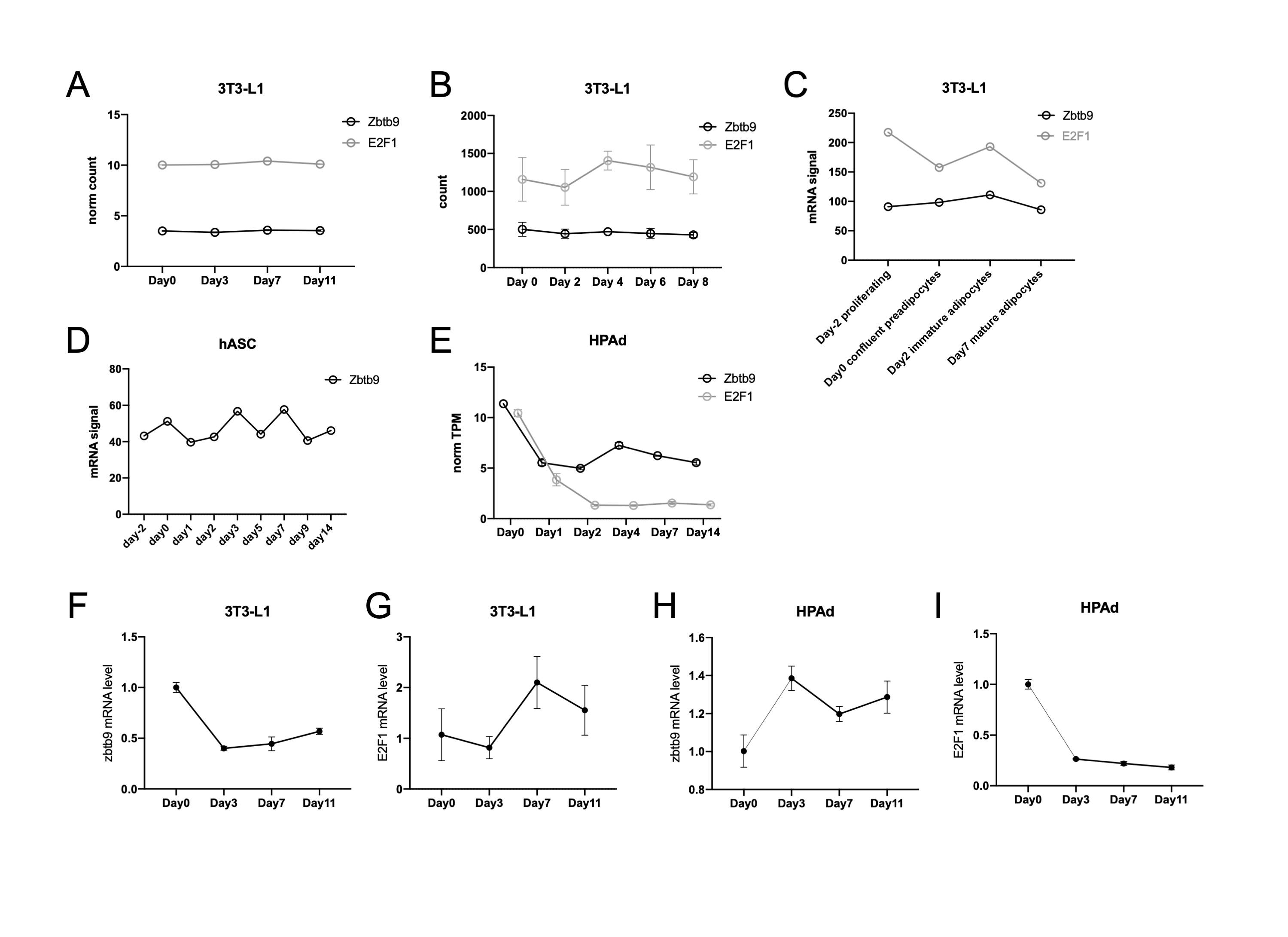


**Figure S3. *Zbtb9* and *E2F1* gene expression during adipogenesis.** (A) Normalized count data of *Zbtb9* and *E2F1* in 3T3-L1 shCtrl cells differentiation from our RNA-Seq analysis (GSE253544). (B) Count data of *Zbtb9* and *E2F1* in 3T3-L1 shCtrl cells differentiation by RNA-Seq (GSE95029) (C) *Zbtb9* and *E2F1* mRNA expression in 3T3-L1 cells and (D) human adipose stromal cells (hASC) differentiation by Array (GSE20752) (E) *ZBTB9* and *E2F1* transcripts per million (TPM) in primary human preadipocyte (HPAd) differentiation by RNA-Seq (GSE249195). (F) *Zbtb9* and (G) *E2F1* mRNA levels in 3T3-L1 cells during differentiation as measured by RT-qPCR relative to the control gene *Gapdh*. (H) *Zbtb9* and (I) *E2F1* mRNA level in human preadipocytes during differentiation by RT-qPCR relative to the control gene *GAPDH*.


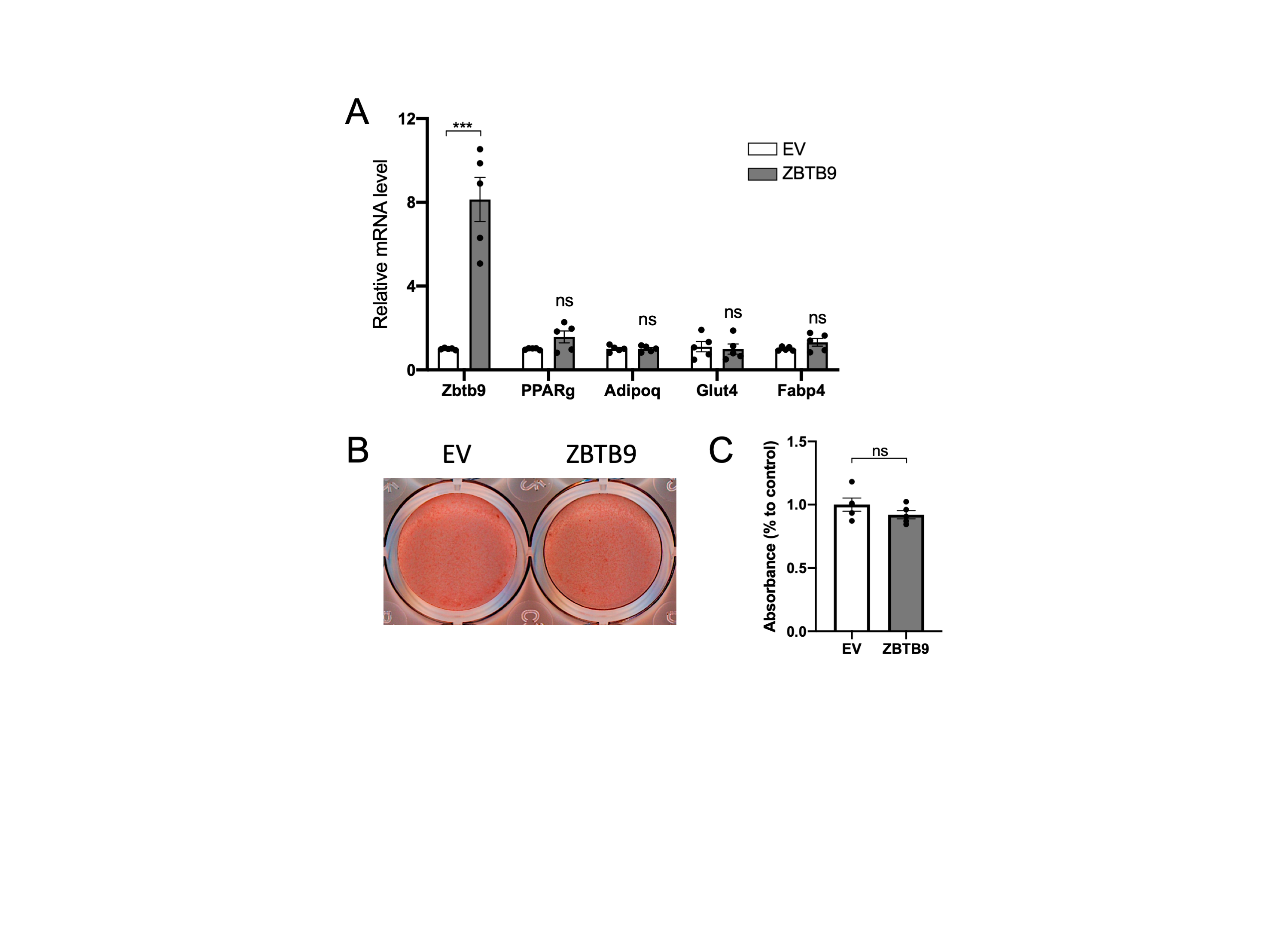


**Figure S4. Zbtb9 overexpression in 3T3-L1 cells has no effect on adipogenesis.** 3T3-L1 preadipocytes stably transduced with the empty vector (EV) or ZBTB9 over-expression vector (ZBTB9) were induced to differentiate. (A) *Zbtb9* and adipogenic gene expression at the end of differentiation (day 7) in the cells as determined by qRT-PCR. (B) Oil Red O staining and (C) quantification to assess lipid accumulation in mature adipocytes.


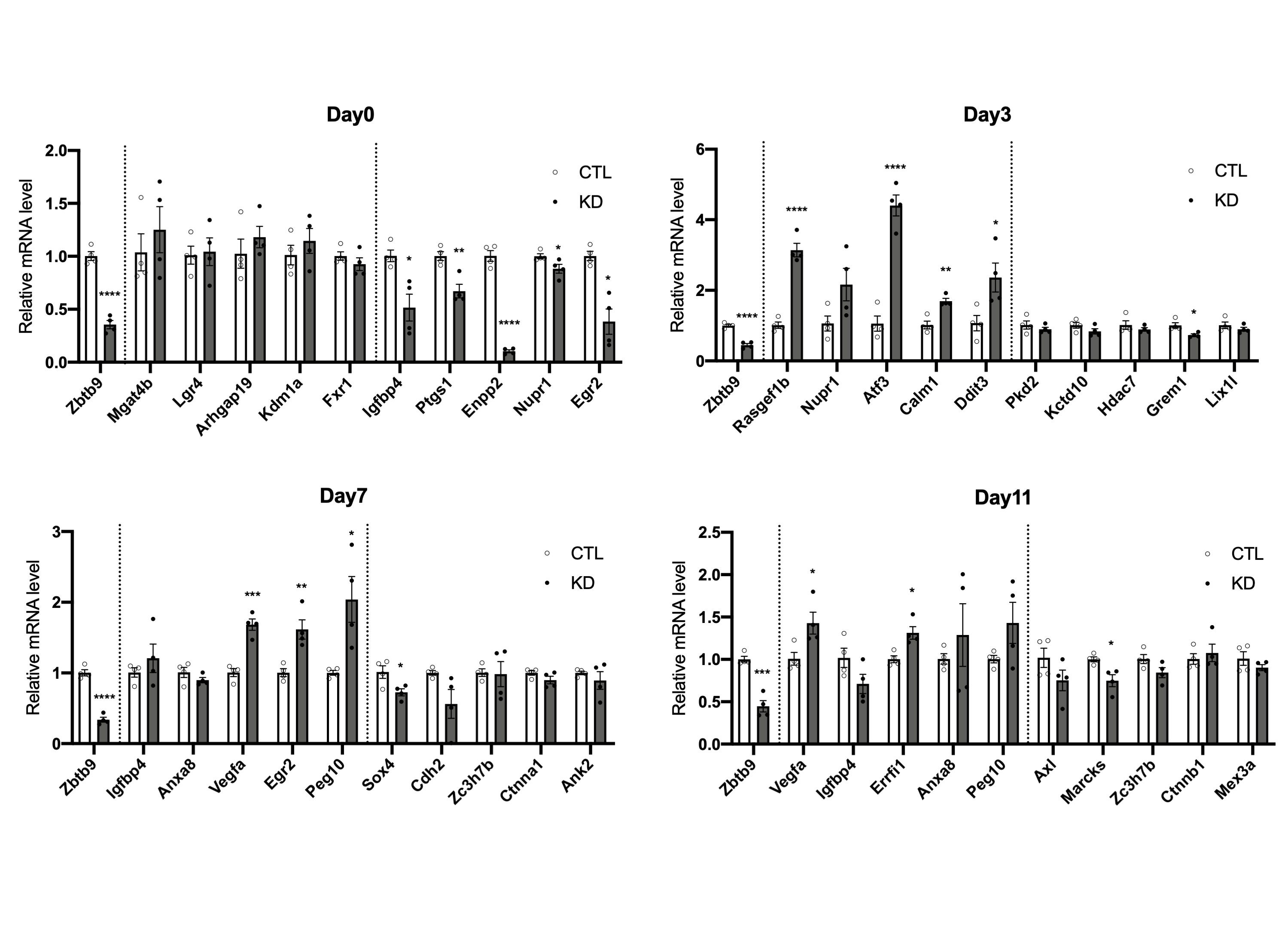


**Figure S5. Validation of DEGs from RNA-Seq.** To validate the results of RNA-Seq analysis, qRT-PCR was performed on independent samples of *Zbtb9*-KD 3T3-L1 cells (*n*=4) and control cells (*n*=4) during differentiation. Gene expression of *Zbtb9* (left), and the top 5 DEGs upregulated (middle) or downregulated (right) at each time point were measured. *p* is not significant unless indicated. * p < 0.05, ** p < 0.01, *** p < 0.001, **** p < 0.0001.

**
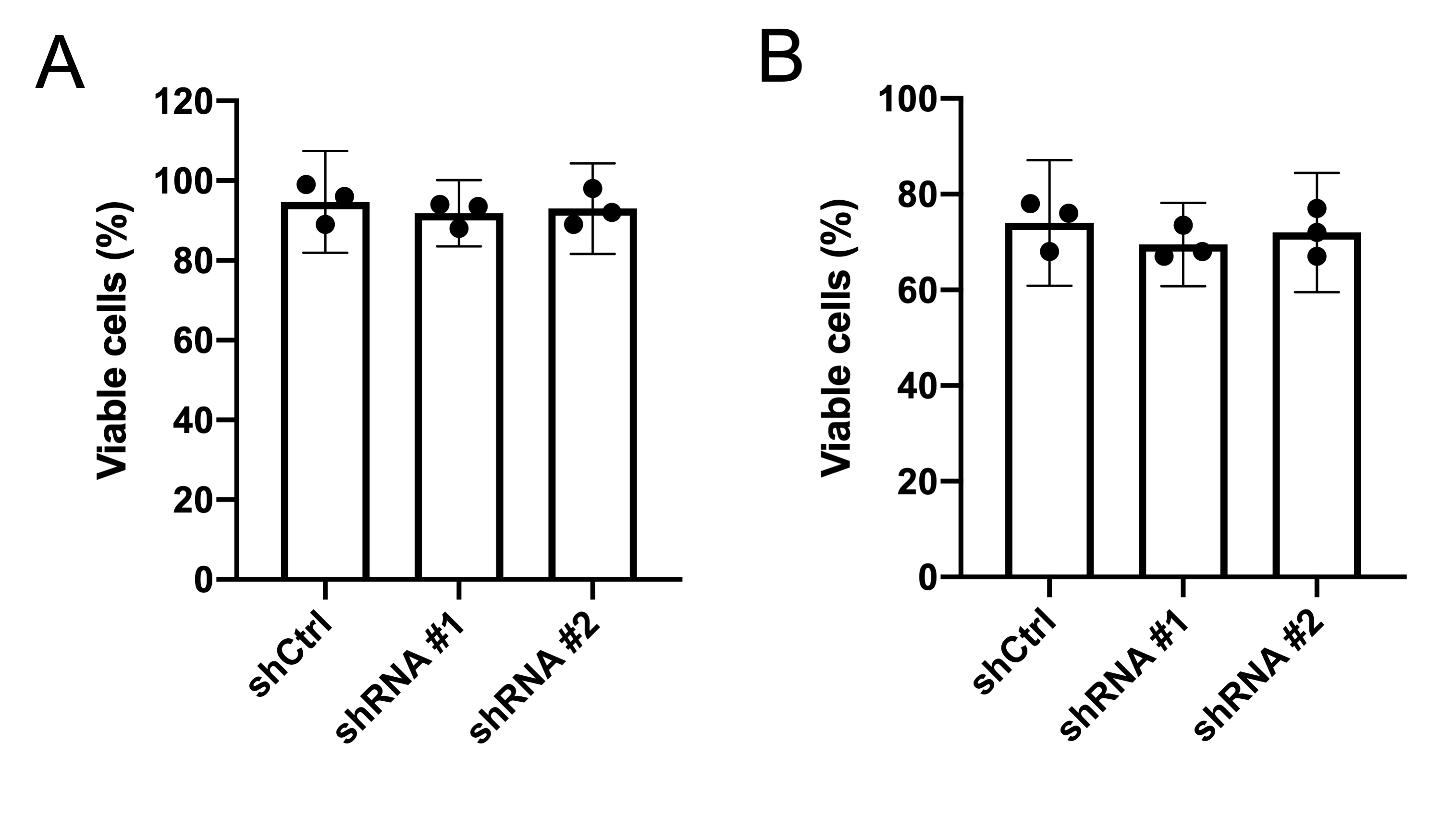
**

**Figure S6. Cell viability in lentivirus transduced cells.** (A) Lentivirus transduced 3T3-L1 preadipocytes and (B) 3T3-L1 mature adipocytes were selected by puromycin for 2 days at which time point no cells not treated with the lentivirus survived. Cells were then stained with trypan blue (0.04%) and blindly counted as positive (dead) or negative (viable). Mean ± 95% confidence intervals are shown. *n* = 121, 154, 209 cells, respectively in preadipocytes. *n* = 92, 74, 130 cells, respectively in mature adipocytes.
